# The Use of Bumper Wire Technique and Intravascular Ultrasound for Precise Aorto-Ostial Stenting

**DOI:** 10.3389/fcvm.2022.929472

**Published:** 2022-07-14

**Authors:** Pavan K. V. Reddy, Joseph Daibes, Michel Skaf, Roberto Ochoa, Tomohiro Fujisaki, Patricia Lin, Apurva Patel, Tak Kwan

**Affiliations:** ^1^Division of Cardiovascular Medicine, Icahn School of Medicine, Mount Sinai Morningside, New York, NY, United States; ^2^Department of Medicine, Icahn School of Medicine, Mount Sinai Morningside and West, New York, NY, United States; ^3^Chinatown Cardiology, New York, NY, United States

**Keywords:** aorto ostial, intravascular ultrasound (IVUS), intervention, Coronary Artery Disease, stent

## Abstract

**Background:**

Aorto-ostial interventions are challenging due to the limitations of contemporary equipment, imprecise ostial demarcation, and problematic ostial lesion characteristics. Suboptimal stent placement is common and portends worse clinical outcomes. Procedural and long-term outcomes of the bumper wire technique with intravascular ultrasound (IVUS) assessment have not been investigated.

**Methods:**

A single-center retrospective study was conducted. Patients who underwent ostial lesion percutaneous coronary intervention (PCI) with the bumper wire technique between January 2019 and September 2020 were identified. The primary endpoint was to determine the geographic miss rate defined by inadequate ostial coverage or excess stent protrusion of > 2 mm by IVUS or angiography. The secondary endpoint was target lesion failure (TLF) at 6 months after PCI, defined as the composite of cardiovascular death, target vessel myocardial infarction (MI), and target lesion revascularization.

**Results:**

In total, 45 patients were identified. The average age was 71.7 years old, and 85.4% were men. Indication for PCI was acute coronary syndrome in about a third of patients. Twenty-six patients had left main ostial lesions and 19 patients had right coronary artery ostial lesions. Geographic miss was detected in two patients (4.4%): one patient (2.2%) had excess proximal stent protrusion and one patient (2.2%) had an ostial miss. Six patients were lost to follow-up. TLF, stroke, or major bleeding were not observed in any of the patients.

**Conclusion:**

The bumper wire technique is safe and efficient with low rates of geographic miss or adverse clinical outcomes. This is the first study to confirm precise aorto-ostial stent implantation with the bumper wire technique using IVUS confirmation.

## Introduction

Aorto-ostial interventions present unique challenges to operators. Technical issues related to equipment include difficulty with guide catheter engagement, device delivery, and pressure dampening (1). Problematic morphologic features of ostial lesions include fibrosis, calcification, and muscular elastic tissue, which makes lesion preparations difficult and predisposes the vessel to recoil (2–4). Additionally, the irregular shape of the coronary ostium and limitations of 2D angiographic imaging cause imprecision in ostial demarcation (5, 6).

“Geographic miss,” defined as incomplete ostial lesion coverage or excess proximal stent protrusion, is common after aorto-ostial interventions and is associated with adverse cardiovascular events (7–9). Several techniques and devices have been developed to reduce geographic miss (10). Intravascular ultrasound (IVUS), in addition to its touted ability to detect stent under-expansion and malapposition, can be aptly utilized to confirm post-implantation ostial coverage and is associated with improved clinical outcomes for aorto-ostial interventions (11). The bumper wire technique, also known as the floating or sepal wire technique, employs a second guidewire placed in the aortic root to mark the ostium and prevent prolapse of the guide catheter past the target ostial lesion. Its use has been described in the literature and is likely utilized regularly in daily practice. However, procedural and long-term clinical endpoints have not been assessed using the gold standard of post-implant IVUS to determine technical success (12, 13). Therefore, this study aims to objectively assess the rate of geographic miss using the bumper wire technique, as well as analyze the long-term clinical outcomes.

## Materials and Methods

This study was approved by the Program for Protection of Human Subjects at the Icahn School of Medicine at Mount Sinai. The approval included a waiver of informed consent.

### Study Design

A single-center retrospective cohort analysis was conducted to investigate procedural success and long-term outcomes of consecutive patients who underwent ostial lesion percutaneous coronary intervention (PCI) with the bumper wire technique at Mount Sinai Morningside Hospital and Mount Sinai Beth Israel Hospital from January 1, 2019, through September 30, 2020. Patients were identified using electronic health records and chart abstraction was completed to provide clinical data, including relevant data points, such as age, gender, ethnicity, presenting symptoms, physical examination, laboratory results, echocardiogram results, coronary angiogram results, diagnosis, treatment regimens, and outcome events. Coronary angiogram and IVUS results were reviewed by two authors (JD and PR). When a consensus was not reached between the two authors, other authors (AP and TK) were consulted to reach a decision. Disagreements were resolved by consensus. The long-term outcomes regarding the safety and efficacy of the bumper wire technique were investigated. All-comers, in which the bumper wire technique was employed, were included in this study, i.e., no eligible cases were excluded.

**FIGURE 1:**
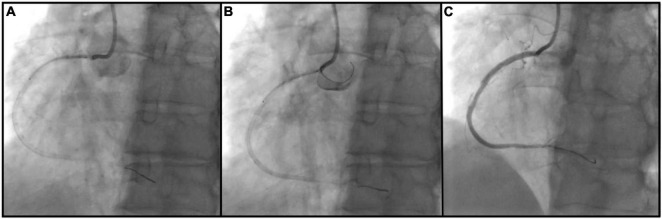
Bumper wire technique procedural steps. **(A)** Ostial right coronary artery lesion is crossed with an intervention wire and coronary stent is positioned outside of the guiding catheter; **(B)** guiding catheter is removed from the ostium and second workhorse wire (bumper wire) is placed in the right aortic cusp with a loop. Steady forward pressure is kept on the guiding catheter which is prevented from coronary intubation by bumper wire. Proximal stent marker is placed at the tip of the guide catheter and the stent is deployed; **(C)** ostial coverage is confirmed in multiple angiographic views.

The bumper wire technique was performed in the following manner (Figure 1): Radial or femoral access was obtained with a standard guiding catheter (typically a 6F or 7F Judkins right (Boston Scientific Corporation, Massachusetts, United States) or Extra Backup (Medtronic, Inc., Minnesota, United States) is advanced to the coronary ostium. A workhorse wire (Runthrough, Terumo, Inc., Tokyo, Japan) is advanced past the lesion. A second guide wire (the bumper wire), is advanced to the guiding catheter tip, and the guide is then removed from the ostium. Following this, the bumper wire is placed with a loop into the coronary cusp. Steady forward pressure is asserted on the guide, which is prevented from intubating the coronary artery by the bumper wire. Then, the stent is loaded onto the coronary wire and placed over the ostial lesion. The stent marker is placed just at the tip of the guiding catheter. Ostial coverage is confirmed in multiple angiographic views aided by ostial demarcation from the bumper wire. The stent is deployed in a normal atmosphere. Then, the stent balloon is withdrawn and deployed in a higher atmosphere to flare up the stent ostium. The bumper wire is then taken out and the guide advanced without damaging the stent. Post-dilation is performed with a high-pressure non-compliant balloon and IVUS is utilized to confirm expansion and proper ostial coverage.

IVUS was utilized in all cases for assessment of lesion coverage and stent protrusion, as well as stent optimization. All cases were done using Eagle Eye Platinum IVUS catheter (Philips, Amsterdam, Netherlands). Under-expansion in left main interventions was determined to be present if the “5-6-7-8 rule” was not met at the case end; a minimum lumen area (MLA) of 5 mm^2^ was required at the LCx ostium, 6 mm^2^ at the LAD ostium, 7 mm^2^ at the point of confluence and 8 mm^2^ in the left main proximal to the polygon (14). For the right coronary ostial lesions, under-expansion was deemed present if MLA was not > 5 mm^2^ or at least 90% of the MLA at the distal reference segment in accordance with the ULTIMATE Trial (15). Edge dissection was defined as dissection into the media with a length of > 3 mm occurring within 5 mm of the stent. Malapposition was defined as a lack of apposition of all stent struts to the vessel wall.

### Definitions and Study Endpoints

The primary endpoint was to determine the geographic miss rate as defined by inadequate ostial coverage or excess stent protrusion of > 2 mm by IVUS or angiography. Secondary endpoints include target lesion failure (TLF) at 6 months after PCI, defined as the composite of cardiovascular death, target vessel myocardial infarction (TVMI), and clinically driven target lesion revascularization (TLR). All MI’s were considered TVMI unless they could be attributed to another non-target vessel. Clinically driven TLR was defined as angina or ischemia referable to the target vessel requiring repeat PCI or CABG (15). Cardiovascular death, spontaneous MI, periprocedural MI, stent thrombosis, and TLR were defined according to the guidelines set forth by the Academic Research Consortium-2 (16). Secondary outcomes also include the individual components of TVF, as well as all-cause death, stroke (ischemic or hemorrhagic), and bleeding [Bleeding Academic Research Consortium (BARC) type 3 or 5] at 6 months from index PCI.

### Statistical Analysis

Normally distributed continuous variables were reported as means with standard deviation (SD). The other continuous variables were reported as median with an interquartile range (IQR). Categorical variables were expressed as proportions. All statistical analyses were done using Statistical Package for the Social Sciences version 27 (IBM, Armonk, New York).

## Results

### Baseline Clinical Characteristics

A total of 45 consecutive patients underwent PCI using the bumper wire technique and adjunct IVUS use for aorto-ostial lesions between January 1, 2019 and September 30, 2020. Six patients did not have to follow-up data at 6 months. Patient demographics are summarized in Table 1. The average age was 71.4 years old and 85.4% were men. A significant proportion of patients had comorbidities, such as hypertension, dyslipidemia, or chronic kidney disease. Approximately 75% of patients had a history of the previous PCI and 73% had multivessel disease. Indication for PCI was acute coronary syndrome in about a third of patients.

**TABLE 1:** Baseline clinical characteristics (*n* = 45).

Age, y	71.4 ± 9.7
Female	7 (15.6)
Race	
White	6 (13.3)
Black	6 (13.3)
Hispanic	9 (20.0)
Asian	23 (51.1)
Other	1 (2.2)
Weight, kg	71.8 (± 17.7)
BMI, kg/m2	26.3 (± 6.2)
Diabetes mellitus	25 (56.0)
Hypertension	41 (91.1)
Dyslipidemia	40 (88.9)
Tobacco use (active or former)	20 (44.4)
Prior CVA	7 (15.6)
Atrial fibrillation	8 (17.8)
Prior myocardial infarction	13 (28.9)
Prior PCI	34 (75.6)
Previous coronary artery bypass grafting	7 (15.6)
Congestive heart failure	19 (42.2)
Left ventricular ejection fraction, %	45.2 ± 15.5
Chronic kidney disease	37 (82.2)
Creatinine, mg/dL	1.6 ± 1.6
eGFR, ml/min/1.73 m^2^	61.2 ± 32.0
Indication for PCI	
STEMI	3 (6.7)
NSTEMI/unstable angina	11 (24.4)
Stable angina/elective	31 (68.9)

*Values are n (%) or mean ± standard deviation. BMI, indicates body mass index; eGFR, estimated glomerular filtration rate; NSTEMI, non-ST-elevation myocardial infarction; PCI, percutaneous coronary intervention; STEMI, ST-elevation myocardial infarction.*

### Procedural Characteristics

The summary of procedural data is shown in Table 2. Approximately two-thirds of the patients had left main ostial lesions and one-third had right coronary artery ostial lesions. Most procedures were performed with radial artery access. Patients had complex coronary anatomies with an average syntax score of 24.1. Between 10 and 20% of patients underwent bifurcation stenting, ostial chronic total occlusion stenting, mechanical circulatory support-assisted PCI, or atherectomy. The average contrast volume was 165.2 ml, and the average fluoroscopy time was 28.8 min. Fluoroscopy time was significantly longer for ostial RCA lesions compared to LM lesions (mean difference: 8.1 min, *p* = 0.03), which may be attributed to the higher rate of CTO interventions in the RCA group.

**TABLE 2:** Procedural characteristics.

	All patients (*n* = 45)	Left main (*n* = 26)	RCA (*n* = 19)
Radial artery access	39 (86.7)	20 (76.9)	19 (100.0)
Femoral artery access	6 (13.3)	6 (23.1)	0 (0.0)
Multivessel disease	33 (73.3)	23 (88.0)	10 (52.6)
Syntax score	24.1 ± 14.5	26.8 ± 10.2	18.0 ± 12.6
Bifurcation lesion	8 (17.8)	8 (30.7)	0 (0.0)
Chronic total occlusion	3 (6.7)	0 (0.0)	3 (15.8)
Mechanical circulatory support	5 (11.1)	4 (15.4)	1 (5.3)
Atherectomy	10 (22.2)	6 (23.1)	4 (21.0)
Stent diameter, mm	3.7 ± 0.4	3.9 ± 0.2	3.5 ± 0.5
Stent length, mm	23.1 ± 8.3	22.9 ± 7.9	23.1 ± 9.2
Contrast volume, ml	165.2 ± 69.5	153 ± 57.9	171.1 ± 62.2
Fluoroscopy time, min	28.8 ± 12.5	25.5 ± 11.8	33.5 ± 12.4

*Values are n (%) or mean ± standard deviation. RCA indicates right coronary artery.*

**FIGURE 2:**
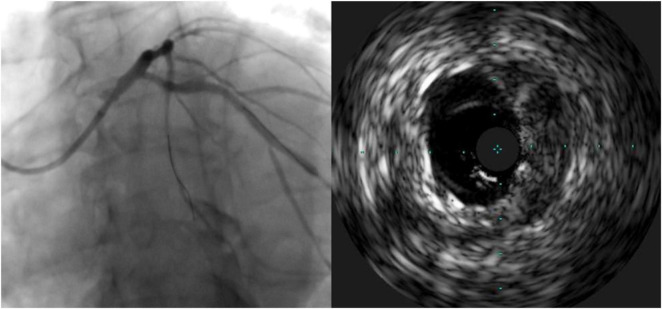
Case of geographic miss showing incomplete ostial coverage by angiography and intravascular ultrasound.

Table 3 demonstrates the geographic miss rate and final adverse IVUS findings after stent optimization was attempted, i.e., suboptimal stent implantation. Geographic miss was detected in two patients (4.4%): one patient (2.2%) had more than 2 mm of excess stent proximal protrusion and one patient (2.2%) was found to have an ostial miss, which required an additional ostial stent (Figure 2). One case (2.2%) of under-expansion was left unresolved.

**TABLE 3:** Procedural characteristics: geographic miss and intravascular ultrasound findings.

	All patients (*n* = 45)	Left main (*n* = 26)	RCA (*n* = 19)
**Geographic miss**	2 (4.4)	2 (7.7)	0 (0.0)
Excess stent proximal protrusion (> 2 mm)	1 (2.2)	1 (3.8)	0 (0.0)
Ostial miss	1 (2.2)	1 (3.8)	0 (0.0)
**IVUS findings after optimization**			
Under-expansion	1 (2.2)	1 (3.8)	0 (0.0)
Malapposition	0 (0.0)	0 (0.0)	0 (0.0)
Edge dissection	0 (0.0)	0 (0.0)	0 (0.0)

*Values are n (%). IVUS indicates intravascular ultrasound. RCA indicates right coronary artery.*

### Clinical Outcomes at 6 Months

A summary of clinical outcomes is shown in Table 4. Six patients failed to follow-up 6 months after the procedure. TLF was not observed in any patients, for which follow-up data was available. Secondary outcomes were notable for one non-cardiovascular death and three patients with type 2 MI. No stroke or BARC 3 or 5 bleeding was observed.

**TABLE 4:** Clinical outcomes at 6-month follow-up.

Clinical outcome	All patients (*n* = 39)	Left main (*n* = 22)	RCA (*n* = 17)
TLF	0 (0.0)	0 (0.0)	0 (0.0)
CV Death	0 (0.0)	0 (0.0)	0 (0.0)
TVMI	0 (0.0)	0 (0.0)	0 (0.0)
TLR	0 (0.0)	0 (0.0)	0 (0.0)
Myocardial infarction	3 (7.7)	2 (9.1)	1 (5.9)
All-cause death	1 (2.6)	1 (4.5)	0 (0.0)
Stent thrombosis	0 (0.0)	0 (0.0)	0 (0.0)
CVA	0 (0.0)	0 (0.0)	0 (0.0)
Bleeding	0 (0.0)	0 (0.0)	0 (0.0)

*Values are n (%). CV, indicates cardiovascular; CVA, cerebral vascular accident; RCA, right coronary artery; TLF, target lesion failure; TLR, target lesion revascularization; TVMI, target vessel myocardial infarction.*

## Discussion

This single-center experience confirms the efficacy and safety of the bumper wire technique for precise stent implantation in aorto-ostial interventions. This is the first study utilizing IVUS to demonstrate the rare occurrence of inadequate lesion coverage or excess proximal stent protrusion when employing the bumper wire technique. We also demonstrated that adverse clinical outcomes at 6 months were very low, limited to one non-cardiovascular death and three instances of MI unrelated to target vessel intervention while TLR or TVMI was not seen.

Aorto-ostial interventions have been associated with worse clinical outcomes when compared to non-ostial interventions, possibly due to high rates of geographic miss along with the tendency of ostial lesions to be unyielding. The occurrence of geographic miss among aorto-ostial interventions is excessively high and vastly underestimated by angiography alone. Rubinshtein et al. analyzed aorto-ostial stents with coronary computed tomography angiography and discovered a geographic miss in 87% of cases despite decidedly optimal stent position by angiography in 95% of those cases (5). Another study reported a geographic miss in 54% of cases, with a similar incidence of distal and proximal miss (6). These findings underscore the need to employ specialized techniques above conventional methods. Many devices and methods have been described for this purpose, including the Szabo (tail-wire, buddy wire) technique, the Ostial Pro device (Ostial Solutions, Kalamazoo, MI, United States), and the Flash Ostial system (Ostial Corporation, Sunnyvale, CA) (17–19). The Szabo technique is effective but requires a long learning curve and involves an elevated risk of stent deformation or loss (20–22). The Ostial Pro has also been shown to be effective in reducing geographic miss and is relatively user-friendly, but it requires the lab to stock an extra device. The bumper wire technique entails several advantages over other methods, including technical ease, as well as cost and time efficiency. Results from this study indicate that the procedure success rate is excellent regarding precise ostial stenting (4.4% geographic miss), with an exceedingly low event rate at 6 months (0 cases of TLF) in a small sample.

To our knowledge, this is the first study to confirm precise aorto-ostial stent implantation by IVUS after intervention with the bumper wire technique. Taştan et al. described procedural and clinical advantages of the bumper wire technique when compared to the conventional method. However, this study did not use IVUS to confirm ostial coverage, and left main interventions were not included. The use of IVUS to guide aorto-ostial intervention is supported by retrospective evidence and advocated for by a consensus statement (1, 23). Patel et al. reviewed aorto-ostial interventions and found that IVUS use was associated with significantly lower rates of MI and TLR when compared to a cohort without IVUS use. The potential mechanism of benefit may be due to a demonstration of stent under-expansion in 40% and suboptimal lesion coverage in 10%, which led to further optimization in 56% of cases (11). Low clinical event rates in the current study may be related to the routine use of IVUS with the limited occurrence of suboptimal stenting (one case of stent under-expansion). Importantly, there was one occurrence of inadequate ostial coverage appreciated by IVUS which was not readily discernible by angiography alone (Figure 2). If left untreated, this residual ostial stenosis would be predisposed to angina recurrence, restenosis, and potentially stent thrombosis.

Several limitations should be considered when making conclusions from this study. This is a retrospective, single-center experience, and, therefore, commonly recognized shortcomings of these two aspects should be acknowledged. While this study is one of few to include both left and right coronary interventions, evidence for use of the bumper wire technique in ostial graft interventions is still lacking (24, 25). This study did not utilize a comparator group due to a lack of equipoise among operators, thus, limiting the interpretation of observed clinical outcomes without a reference population. However, the event rate was objectively very low even with a high comorbidity rate, large coronary disease burden (73% with multivessel disease), and complex interventions often entailing atherectomy, bifurcation stenting, chronic thrombotic occlusions, and mechanical circulatory support.

## Conclusion

In conclusion, the bumper wire technique for aorto-ostial intervention is safe and effective, with low rates of geographic miss seen by IVUS and with rare adverse clinical outcomes.

## Data Availability Statement

The raw data supporting the conclusions of this article will be made available by the authors, without undue reservation.

## Author Contributions

PR, TF, AP, and TK made substantial contributions to the study design and development of the manuscript. PR, JD, MS, RO, and TF performed data collection. PL completed statistical analysis. TK had been identified as the guarantor of the manuscript, taking responsibility for the integrity of the work as a whole, from inception to published article. All authors contributed to the article and approved the submitted version.

## Conflict of Interest

The authors declare that the research was conducted in the absence of any commercial or financial relationships that could be construed as a potential conflict of interest.

## Publisher’s Note

All claims expressed in this article are solely those of the authors and do not necessarily represent those of their affiliated organizations, or those of the publisher, the editors and the reviewers. Any product that may be evaluated in this article, or claim that may be made by its manufacturer, is not guaranteed or endorsed by the publisher.

## Abbreviations

BARC: Bleeding Academic Research Consortium
IVUS: Intravascular ultrasound
MI: Myocardial infarction
PCI: Percutaneous coronary intervention
TLF: Target lesion failure
TLR: Target lesion revascularization
TVMI: Target-vessel myocardial infarction.
